# Pulmonary Manifestations of Primary Humoral Deficiencies

**DOI:** 10.1155/2022/7140919

**Published:** 2022-04-10

**Authors:** Ana Casal, Vanessa Riveiro, Juan Suárez-Antelo, Lucía Ferreiro, Nuria Rodríguez-Núñez, Adriana Lama, María Elena Toubes, Luis Valdés

**Affiliations:** ^1^Pneumology Department, Hospital Clínico Universitario, Santiago de Compostela, Spain; ^2^Pneumology Interdisciplinary Research Group, Santiago de Compostela Health Research Institute (IDIS), Santiago de Compostela, Spain; ^3^University of Santiago de Compostela, Santiago de Compostela, Spain

## Abstract

Primary immunodeficiencies are a group of conditions characterized by developmental or functional alterations in the immune system caused by hereditary genetic defects. Primary immunodeficiencies may affect either the innate or the adaptive (humoral and cellular) immune system. Pulmonary complications in primary humoral deficiencies are frequent and varied and are associated with high morbidity and mortality rates. The types of complications include bronchiectasis secondary to recurrent respiratory infections and interstitial pulmonary involvement, which can be associated with autoimmune cytopenias, lymphoproliferation, and a range of immunological manifestations. Early detection is key to timely management. Immunoglobulin replacement therapy reduces the severity of disease, the frequency of exacerbations, and hospital admissions in some primary humoral deficiencies. Therefore, the presence of pulmonary disease with concomitant infectious and/or autoimmune complications should raise suspicion of primary humoral deficiencies and warrants a request for immunoglobulin determination in blood. Once diagnosis is confirmed; early immunoglobulin replacement therapy will improve the course of the disease. Further studies are needed to better understand the pathogenesis of pulmonary disease related to primary humoral deficiencies and favor the development of targeted therapies that improve the prognosis of patients.

## 1. Generalities about Primary Immunodeficiencies

Primary immunodeficiencies (PIDs) are caused by a deficient development or function of the immune system secondary to an often hereditary genetic defect. PIDs affect the innate as well as the adaptive (humoral and cellular) immune system. In general terms, PIDs and, above all, primary humoral deficiencies (PHDs), are associated with a higher risk for infections, autoimmune diseases, and neoplasms; therefore, early diagnosis is crucial. PIDs comprise more than 250 syndromes, of which, only a group is clinically relevant.  Table 1 details the most frequent PIDs.

Immunoglobulins (Ig) play a key role in protecting the lungs from infections, with predominance of a range of specific isotypes in the different parts of the airways [1]. At the level of the airways, the most frequent Igs are IgA and IgM, whereas IgG is more commonly found in the alveolar space [2]. IgA prevents bacterial adhesion and neutralizes toxins, whereas IgM activates the complement system, which allows the opsonization of pathogens. In turn, IgG (originated from the systemic circulation) enters the airways through passive diffusion and provides protection against infections, such as pneumonia [3]. In most patients with PHDs, systemic IgG and local IgA are absent or dysfunctional, and serum IgA compensates, at least partially, IgG deficiency in the airways.

### 1.1. Physiopathological Mechanisms of Primary Humoral Deficiencies

The development of PHDs is mediated by a range of physiopathological mechanisms. Some PHDs are caused by developmental defects in memory B cells result in the loss of Igs production [*i.e: X-linked agammaglobulinemia (XLA)*]. Other patients produce Igs but at low trough levels, which compromises protection against infections [*i.e.: common variable immunodeficiency (CVID)*]. In some cases, patients produce normal IgM values but are unable to perform class-switch recombination (*i.e.: hyper-IgM syndrome*). Other patients do not produce IgG subclasses (*i.e.: IgG-subclass deficiency*). Finally, other subjects do not develop Ig specific enough to neutralize a specific pathogen (i.e.: *specific antibody deficiency*) [4].

## 2. Pulmonary Complications Associated with Primary Humoral Deficiencies

Lung disease is a frequent complication of PHDs, with high morbidity and mortality rates. The range of clinical manifestations is broad, including acute and chronic infectious processes, structural abnormalities, and malignancies (Table 2). The chronic lung diseases most commonly associated with PHDs are bronchiectasis and interstitial lung disease (ILD). Whereas bronchiectasis result from recurrent respiratory tract infections, susceptibility to ILD is determined by multiple factors and can be induced by processes such as cytopenias, autoimmune diseases, lymphoproliferative syndromes, and/or immune dysregulation.

### 2.1. Respiratory Infections

The main clinical implication of PHDs are recurrent bacterial infections in the upper and lower airways [5]. There is a high risk for recurrent pneumonia, which has been reported in 75–84% of patients with CVID [6]. The most frequent causative agents include encapsulated bacteria (*Haemophilus influenzae* or *Streptococcus pneumoniae*). Recurrence and rare complications such as pneumatoceles and cavitation should raise suspicion of an underlying immunodeficiency. Early diagnosis and management with prophylactic antibiotics and Ig replacement therapy reduce the frequency of infections and their long-term effects (chronic airflow obstruction, atelectasis, and bronchiectasis, to name a few) [7].

### 2.2. Bronchiectasis

Bronchiectasis is the most frequent chronic airway disease associated with PHDs, and it is reported in 30–60% of patients with CVID [8]. This condition is also common in IgG subclass deficiency, especially when associated with IgA deficiency. PHDs increase the frequency of pulmonary infections that characterize bronchiectasis. Bronchiectasis derives from long-standing active inflammation resulting from recurrent infections, which causes irreversible dilatation of the bronchial airways and perpetuates respiratory tract infections (Cole's vicious circle) [9]. A similar vicious circle may occur in chronic sinusitis. Immune defects are considered relevant factors in the development of bronchiectasis.

As stated before, milder forms of PHDs (lower levels of IgA or IgM) are also associated with bronchiectasis, which potentially may progress. For this reason, IGRT should be considered to minimize chronic respiratory complications and improve prognosis [10].

Patients usually develop dyspnea, prolonged cough, purulent sputum, and, occasionally, hemoptysis. At functional level, variable airflow obstruction, which can be progressive, is observed [11]. Diagnosis is typically based on computed tomography (CT), as chest radiographs can be inadequate. In this line, a recent study found that cough was more associated with bronchiectasis on CT than airflow spirometry obstruction in PHDs patients [12]. Furthermore, colonization of the airways can worsen the course of disease. Screening for sputum colonization is useful for selecting antibiotic therapy and is of vital importance to limit resistance associated with macrolide therapy [13].

Therefore, early diagnosis and treatment are crucial to prevent the deleterious effects of PHDs on the lungs. A gold-standard therapy for adult non-cystic fibrosis (CF) bronchiectasis is not available and is usually extrapolated from CF trials. Physiotherapy is considered a standard therapy, but there are no guidelines defining the best approach. Azithromycin, apart from its antibacterial power, is known to have immunomodulatory effects in chronic lung disorders. Its use has been suggested for its interesting impact on respiratory exacerbations [14]. Despite all this, some patients, however, will still develop bronchiectasis even after Ig replacement therapy and/or associated antibiotic treatment.

### 2.3. Interstitial Lung Disease (ILD)

ILD in patients with PHDs has a distinctive clinical and immunological profile that does not necessarily involve the presence of bronchiectasis. Around 10–20% of patients with CVID develop ILD. ILD appears far less common in other forms of PIDs. The etiology of this disease may be related to infections (human herpes virus 8 or Epstein–Barr virus) or to an underlying immune dysregulation. Differentiating ILD from other forms of chronic lung disease on the basis of the clinical history alone can be challenging, as its most common symptoms are nonspecific. Physical examination may be useful, with crackles being more suggestive of ILD and wheezing being more likely to be with a sign of obstructive disease. The most frequent radiological findings include the presence of pulmonary nodules, ground-glass opacities, and hilomediastinal adenopathies [11]. Diagnosis is based on histological results that exclude other causes (primarily a malignancy such as lymphoma). The range of histological forms of interstitial is broad, being the most common organizing pneumonia, lymphoid interstitial pneumonia, follicular bronchiolitis, or granulomatous disease. Histological patterns may overlap and are not correlated with a particular immune deficiency. Moreover, many types of PHDs are associated with an increased risk for systemic autoimmune disorders that may involve respiratory interstitial tissue (as connective tissue diseases or vasculitis) [4].

Timing treatment for ILD is challenging. It is imperative that immunoglobulin replacement therapy is optimized in patients with ILD. The reason is that ILD may stabilize with this intervention in a subset of patients. A reasonable option in patients with mild to moderate ILD symptoms is inhaled corticosteroids (with or without long-acting beta agonists) and/or prophylactic azithromycin, as these therapies have been shown efficacious in follicular bronchiolitis [15]. Systemic corticosteroid treatment and/or immunomodulators may also be effective.

### 2.4. Granulomatosis

The presence of non-necrotizing granuloma in a lung tissue specimen is nonspecific and may be associated with infections or other types of processes (drugs, interstitial disease, to name a few). Up to 25% of patients with CVID develop non-necrotizing granuloma as a manifestation of granulomatous-lymphocytic interstitial lung disease (GLILD). This occasionally leads to misdiagnosis of sarcoidosis due to their radiological and histological similarities (Table 3). Therefore, serum Ig determination is recommended in all patients with a recent diagnosis of sarcoidosis. Some authors consider GLILD as a pulmonary manifestation of a systemic syndrome associated with autoimmune cytopenias, splenomegaly, enteritis, and lymphadenopathies [16]. Definitive diagnosis requires histological confirmation by a surgical lung biopsy. Treatment involves high doses of corticosteroids, although the disease may progress despite optimal treatment [17]. There is limited evidence demonstrating that the use of rituximab associated with azathioprine may improve lung function and/or radiological findings in patients with GLILD [18, 19].

### 2.5. Obliterative Bronchiolitis

As previously mentioned, long-standing inflammation resulting from recurrent respiratory tract infections may induce a progressive narrowing of the bronchial lumen, thereby causing obliterative bronchiolitis. This may also occur in rheumatological diseases frequently associated with PHDs. The presence of disproportionate chronic airflow obstruction for the degree of bronchiectasis, reduced DLCO, and/or oxygen desaturation with exertion in a patient with immunodeficiency are suggestive of obliterative bronchiolitis [20].

### 2.6. Respiratory Amyloidosis

Amyloidosis is characterized by extracellular deposition of insoluble misfolded proteins that aggregate in tissues and cause organ dysfunction [21]. There are multiple forms of amyloidosis, and diagnosis requires histological confirmation. Light chain amyloidosis (AL) most frequently affects the lungs, and it may affect any of its structures. Respiratory complications are less frequent in other forms of amyloidosis, such as serum amyloidosis (SAA) [22]. Systemic amyloidosis does not only affect the lungs (parenchyma, adenopathies, and pleura), but it may also involve other organs, such as the kidneys and/or the heart. In contrast, localized amyloidosis only affects the respiratory tract (nodules, cysts, and ILD).

Tracheobronchial amyloidosis accounts for 1.1% of cases [23], presents in patients older than 60 years, shows no gender-based differences, and may appear in systemic forms of amyloidosis [24]. Associated chronic airflow obstruction may lead to misdiagnosis of asthma or chronic obstructive pulmonary disease. Findings on chest CT scan include a narrowing of the bronchial lumen, atelectasis, and/or bronchiectasis [25]. Definitive diagnosis requires histological confirmation. Prognosis is poor and depends on the frequency of respiratory tract infections and progressive airflow obstruction.

Nodular and cystic amyloidosis accounts for 44–58% of all respiratory forms and usually appears in patients in their sixties, being more frequent in male subjects (men:women, 3 : 2) [26]. More than half the patients suffer from a related connective tissue disease (prevailingly Sjögren's disease). Pulmonary nodules may be multiple, have different shapes and sizes, and are associated with cysts in up to 81% of cases [27].

Interstitial amyloidosis is generally secondary to an underlying disease (lymphoproliferative or connective tissue disease) and may be constrained to the lung (rarely) or be associated with systemic amyloidosis (predominant form). Specific radiological findings include irregular linear opacities, thickening of interlobular septae, honeycombing, bronchiectasis, nodules, cysts, and growth of hilomediastinal lymph nodes.

Pulmonary vascular amyloidosis is usually associated with systemic amyloidosis involving the heart. In most cases, patients remain asymptomatic, although cases of hemoptiyis, hemothorax, and other hemorrhagic diatheses have been reported [28].

Finally, pleural amyloidosis is a rare form of respiratory amyloidosis and accounts for 5–10% of respiratory forms, being exclusive of AL forms. The resulting pleural effusion is a transudate, an exudate or a chylothorax. It has a poor prognosis and has a median survival of 1.6 months, which decreases with heart involvement [29].

### 2.7. IgG4-Related Pulmonary Disease

IgG4-related lung disease (IgG4-RD) is a chronic fibroinflammatory disorder characterized by lymphoplasmacytic infiltrates, tissue fibrosis, and elevated plasma IgG4 levels [30]. IgG4-RD may present as a solitary lesion (20% of cases) or affect multiple organs (with synchronous or metachronous involvement of two or more extrapulmonary organs). Manifestations of the systemic form include a weight loss of 5–10 kg, pancreatitis, sialadenitis, lymphadenopathy, retroperitoneal/periaortic fibrosis, and/or tubulointerstitial nephropathy, among others [31]. Definitive diagnosis requires histological confirmation. Both, symptomatic and asymptomatic forms require treatment to prevent their potentially irreversible effects. Management involves long-term corticosteroid therapy (maintenance treatment for 1 to 3 years) combined or not with immunosuppressive therapy with corticosteroid-sparing drugs (mycophenolate mofetil, azathioprine, methotrexate, and/or rituximab) [32, 33]. IgG4-RD has an uncertain diagnosis, and relapse is associated with elevated levels of IgG4, IgE and eosinophils [34].

### 2.8. Malignant Diseases Involving the Lung

Neoplastic disorders are a major cause of death in patients with PHDs. CVID patients are at an increased risk of gastric carcinoma and lymphoma [35]. PHDs patients may develop different types of lymphoid lung lesions (B-cell and non-Hodgkin and Hodgkin lymphoma). These should be considered in differential diagnosis of GLILD [36]. Lung metastases or primary carcinoma have also been reported in PHDs [37].

## 3. Types of Primary Humoral Deficiencies

### 3.1. Selective IgA Deficiency

Selective IgA deficiency (SIgAD) is the most frequent PHDs, with an estimated prevalence of 1 : 600 in North America and Europe [38]. Diagnosis is based on serum levels of IgA <7 mg/dL and normal levels of IgG and IgM. SIgAD is caused by a defect in B-cell maturation. Two out of three patients with SIgAD remain asymptomatic, whereas the remainder will develop recurrent bacterial infections, autoimmune diseases, gastrointestinal problems, and/or atopy [39]. The fact that most patients remain asymptomatic suggests the presence of IgA compensatory mechanisms at the level of the local mucosa. Additionally, it is known that symptomatic patients may also present IgG subclass deficiency (primarily IgG2), which is associated with the presence of bronchiectasis [40]. Likewise, there is evidence of a higher frequency of SIgAD in families with a member suffering from CVID, which suggests a relationship between the two conditions or even a potential progression of SIgAD into CVID [41].

### 3.2. Common Variable Immunodeficiency

The estimated prevalence of CVID ranges from 1 : 25.000 to 1 : 50.000, without gender-based differences, and onset generally occurs between the age of 20 and 40 years [42]. CVID is characterized by a reduction in IgG and IgA production, associated or not with IgM, which results in low responsiveness to polysaccharide vaccines. The diagnostic criteria for CVID of the *European Society of Inmunodeficiency* are described in Table 4 [43]. Although the molecular etiology of CVID is unclear, 10–25% of cases have an autosomal dominant inheritance pattern. CVID is associated with recurrent infections (prevailingly sinopulmonary infection by encapsulated and atypical bacteria), autoimmune diseases (hemolytic anemia, thrombocytopenic purpura, thyroiditis, and rheumatoid arthritis), GLILD, splenomegaly, gastrointestinal problems, or even malignant diseases (non-Hodgkin lymphoma and gastric cancer). Multiorganic involvement is frequent [44]. Due to the multiple forms of presentation of the disease, delayed diagnosis is frequent.

### 3.3. Immunoglobulin Subclass Deficiency

As mentioned above, IgG is the most abundant antibody in the systemic circulation, with 4 subclasses distinguished by structural and functional differences, known as isotypes. Manifestations of Igs subclass deficiency include reduced levels of some IgG isotype and normal levels of IgA and IgM (in two determinations separated by at least one month, during a period free of symptoms) [5]. IgG2 deficiency is more frequent in childhood, and IgG4 is found in 8% of the Caucasian population. Igs subclass deficiency may co-occur with SIgAD, which explains the appearance of autoimmune diseases or asthma. Patients with IgG1 and IgG2 may have poor response to polysaccharide vaccines and are more prone to airway infections by encapsulated pathogens (*Streptococcus pneumoniae* and *Haemophilus influenzae*).

### 3.4. Hyper-IgM Syndrome

Hyper-IgM syndrome embraces a group of disorders characterized by normal or elevated levels of IgM and reduced levels of IgG and IgA [45]. The most frequent form of hyper-IgM syndrome (accounting for 70% of all cases) is X-linked hyper-IgM, where T cells lack functional CD40 ligand and cannot signal B cells to switch [46]. This defect predisposes the patient to bacterial respiratory infections and other infections caused by pathogens less frequently associated with humoral immunodeficiencies, such as *Pneumocystis*. This condition is also associated with a higher risk for gastrointestinal infections and autoimmune diseases [47].

### 3.5. Other Rare Primary Immunodeficiencies

Table 5 describes other rare primary immunodeficiencies (humoral and others) and their main characteristics.

## 4. Diagnosis of Primary Humoral Deficiencies

**Figure 1:**
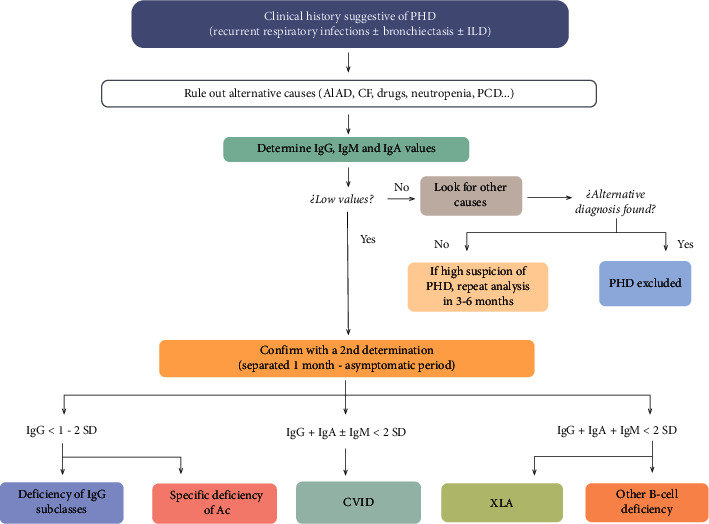
Diagnostic approach to primary humoral deficiencies in patients with respiratory symptoms (modified from Berger et al.) [50]. A1AD, alpha-1-antitrypsin deficiency; Ac, antibodies; CF, cystic fibrosis; CVID, common variable immunodeficiency; ILD, interstitial lung diseases; PCD, primary ciliary dyskinesia; PHD, primary humoral deficiency; SD, standard deviation; XLA, X-linked agammaglobulinemia.

Although the mechanisms of immunodeficiencies are well known, delayed diagnosis is frequent, especially when onset occurs in adulthood. The Immune Deficiency Foundation and the American Academy of Allergy, Asthma and Immunology provide guidelines and establish diagnostic criteria for the appropriate identification of the different syndromes [48, 49]. The first (and most important) step for correct diagnosis is to maintain a high level of suspicion in patients with recurrent infections. In the presence of recurrent infections, respiratory exacerbations, or bronchorrhea, screening, including Ig determination (IgG, IgM and IgA), is recommended. Reduced levels of IgG are suggestive of a primary immunodeficiency.  Figure 1 shows a diagnostic algorithm [50]. It is worth mentioning that normal IgG levels in a patient with recurrent infections does not exclude a diagnosis of PHD, and IgG subclasses and responsiveness to immunization should be assessed.

## 5. Treatment of Primary Humoral Deficiencies

The management of PHDs requires a multidimensional approach. Thus, management includes general measures such as immunization (influenza and pneumococcus), appropriate treatment of infectious complications (appropriate antimicrobial dosage and antibiotic prophylaxis), or immunosuppression (in GLILD), bronchodilators (in case of airway involvement), or hematopoietic stem cell transplantation, where indicated (in combined immunodeficiencies) [5].

The timing of initiation of Ig replacement therapy is controversial. In general terms, the initiation of treatment should be considered in patients with ≥2 hospital admissions in one year or ≥3 recurrent infections managed in ambulatory care. A laboratory analysis should be requested including a hemogram and biochemistry with markers of kidney and liver function, serologies for HIV, HBV, and HCV, and IgA determination (risk for anaphylaxis in the presence of IgA deficiency due to the potential presence of anti-IgA Ab) [11]. Treatment is generally intravenous (monthly) or subcutaneous (weekly), and blood pressure should be monitored during first infusion. The starting dose is an intravenous dose of Ig (400–600 mg/kg/month) to maintain IgG values within the 500–800 mg/dL range. In some cases, it is necessary to increase the therapeutic target to 800–1000 mg/dL to achieve the intended effects [51]. During follow-up, the number of episodes of infection will determine the most appropriate dose [52].

Immunoglobulin replacement therapy reduces the progression of chronic inflow obstruction in patients with recurrent respiratory infections [51]. Immunoglobulin replacement therapy also improves pulmonary function (FEV_1_ and FVC) in patients with respiratory disease, when the levels of serum IgG achieved exceed 500 mg/dL [53].

## 6. Monitoring Primary Humoral Deficiencies

Survival of patients with PHDs has improved in past few decades as a result of prompt diagnosis and adequate treatment [54]. Nevertheless, reducing the number of exacerbations is not always achieved [55]. There are no international guidelines that describe the appropriate monitoring approach for patients with lung involvement secondary to PHDs. It has been suggested that they should be evaluated (lung function, imaging studies, and laboratory tests) at least once every 6–12 months [55]. It has also been recommended to perform a thorax CT scan in treated patients with respiratory symptoms to monitor the potential progression of lung complications [56].

## 7. Conclusions

The range of respiratory manifestations of PHDs is broad, including infections and immunological disorders, which initially are reversible, but may ultimately cause chronic airflow obstruction. Therefore, early detection and management of an underlying PHD in a patient with lung disease is crucial. IGRT reduces the severity and frequency of exacerbations and decreases hospital admissions and their associated costs, especially in some humoral immunodeficiencies. The presence of lung disease with associated infectious and/or autoimmune complications is strongly suggestive of PHD [57]. In this context, it is necessary to determine plasma Ig levels and, where appropriate, provide early Ig replacement therapy to improve patient prognosis [58].

## Figures and Tables

**Table 1:** Most common primary immunodeficiencies and type of associated immune defect.

Primary immunodeficiencies	Type of immunity affected
Selective IgA deficiency	Humoral immunity
Common variable immunodeficiency	Humoral immunity
Immunoglobulin subclass deficiency	Humoral immunity
X-linked agammaglobulinemia	Humoral immunity
Hyper-IgM syndrome.	Humoral immunity
DiGeorge syndrome	Cellular immunity
Severe combined immunodeficiency	Humoral and cellular immunity
Wiskott–Aldrich syndrome	Humoral and cellular immunity
Complement alteration	Innate immunity
Phagocyte dysfunction	Innate immunity
Alteration in pattern recognition receptors	Innate immunity

**Table 2:** Pulmonary complications associated with primary immunodeficiencies.

PIDs group	Frequency	Pulmonary complication Infectious	Example of PIDs	Other
Humoral immunity	50–60%	Recurrent pneumonia Infections by encapsulated or atypical pathogens	Airway disease (bronchiectasis or asthma) ILD (LIP, COP, GLILD)	CVID IgA deficiency XLA
Cellular immunity	5–10%	Recurrent pulmonary infections (*Pseudomonas*, *Pneumocystis jiroveci*, CMV, *Aspergillus*…)	Airway disease (bronchiectasis, BO) Malignancy (lymphoma)	Wiskott–Aldrich DiGeorge
Combined ID (humoral + cellular)	20%	Opportunistic infections in childhood (CMV, P. jiroveci, *Microbacteria*…)	ILD Malignancy (lymphoma, leukemia)	SCID Ataxia telangiectasia
Phagocyte disorders	10–15%	Recurrent pulmonary infections (*S. aureus*, *Klebsiella*, *Serratia*, *Nocardia*, *Aspergillus*…) Necrotizing pneumonia Pulmonary abscess Empyema	Autoimmune disease Antiphospholipid syndrome ILD (granulomatosis)	CGD Chédiak-Higashi
Complement deficiency	2%	Infections by encapsulated pathogens (S. Pneumoniae, H. Influenzae, N. Meningitidis)	Autoimmune disease (vasculitis, SLE)	Complement deficiency

CGD, chronic granulomatous disease; CMV, cytomegalovirus; COP, Cryptogenic organizing pneumonia; CVID, common variable immunodeficiency; GLILD, granulomatous-lymphocytic interstitial lung disease; ID, immunodeficiency; ILD, diffuse interstitial lung disease; LIP, lymphoid interstitial pneumonia; OB, obliterative bronchiolitis; PIDs, primary immunodeficiency; SCID, severe combined immunodeficiency; SLE, Systemic lupus erythematosus; XLA, X-linked agammaglobulinemia.

**Table 3:** Differential characteristics of GLILD *vs* sarcoidosis.

Characteristics:		GLILD - CVID	Sarcoidosis
Laboratory analysis	Immunoglobulin values Autoimmune cytopenias	IgG + IgA ± IgM Autoimmune hemolytic anemia Autoimmune thrombocytopenic purpura	—
Clinical signs		Splenomegaly Recurrent infections	—
Associated ILD		Lymphoid interstitial pneumonia Follicular bronchiolitis	—
BF	CD4/CD8 ratio	Low	High (CD4/CD8: >3,5)
HRCT	Site predominance Nodules Enlarged hilomediastinal lymph nodes Bronchiectasis	Lower >1 cm Random distribution Frequent Frequent	Upper <1 cm Perilymphatic Frequent —
Treatment		Immunosuppression (corticoids ± azathioprine/mycophenolate/rituximab) Immunoglobulin replacement	Immunosuppression (corticoids ± methotrexate *vs* other immunosuppressants)
Prognosis		Poor prognosis	Good prognosis Possibility of spontaneous remission

BAL, bronchoalveolar lavage; CVID, common variable immunodeficiency; GLILD, granulomatous-lymphocytic interstitial lung disease; HRCT, high-resolution computerized tomography; ILD, interstitial lung diseases.

**Table 4:** Diagnostic criteria for common variable immunodeficiency (modified from Seidel et al.)[32].

Required criterion	Values < 2DS of IgG + IgA ± IgM (in 2 tests)
≥ 1 of subsequent tests	Increased susceptibility to infections
	Autoimmune manifestations
	Granulomatous disease
	Unexplained polyclonal lymphoproliferation
	A member of the family suffers from humoral immunodeficiency
≥ 1 of subsequent tests	Poor response to immunization (and/or absence of isohemagglutinins)
	Low levels of memory B cells (<70% of the reference value for the age of the patient)
Required criteria (all)	Exclusion of secondary causes of hypogammaglobulinemia
	Minimum age ≥4 year to establish diagnosis
	Absence of notable T-lymphocytes deficiency

**Table 5:** Rare primary immunodeficiencies and their characteristics.

PIDs	Type of immunity affected	Characteristics
X-linked agammaglobulinemia	Humoral	85% of congenital agammaglobulinemias Caused by mutations in Bruton's tyrosine kinase (BTK) located in the X chromosome Absence of BTK protein: Absence of B-cell differentiation and reduction of all Ig isotypes Susceptibility to bacterial infections from 6 months of age Susceptibility to encapsulated bacteria and viruses
Severe combined immunodeficiency	Humoral and cellular	Heterogeneous group of life-threatening PIDs with dramatic reduction of T and B lymphocytes ± NK cells Caused by a variety of genetic alterations (IL2RG, ADA, IL7R, RAG1, RAG2, JAK3…) Susceptibility to recurrent severe infections, chronic diarrhea, and failure to thrive Treatment includes prophylaxis/management of infections, Ig replacement, and hematopoietic stem cell transplantation
Wiskott-Aldrich syndrome	Humoral and cellular	Hereditary X-linked disorder caused by WAS protein mutations Susceptibility to thrombocytopenia, bacterial/fungal/viral infections, eczema, autoimmune diseases and malignancy (lymphoma) Treatment includes prophylactic antibiotics, platelet transfusions, ig replacement, immunosuppressants, and hematopoietic stem cell transplantation
DiGeorge syndrome	Cellular	Most frequent cause: 22q11.2 chromosome deletion Classic triad: Congenital heart disease, thymic hypoplasia and hypocalcemia Susceptibility to recurrent infections, autoimmunity, developmental delay, cleft palate and chronic inflammatory diseases Diagnosis: Low T-lymphocyte values CD3+ associated with typical manifestations and/or chromosome 22q11.2 deletion
Complement alteration	Innate	High number of complement alterations that may affect the classic (CH50) or alternative pathway (AH50) Susceptibility to infections by encapsulated pathogens Frequently associated with SLE Treatment: Immunization and antimicrobial treatment
Alteration in pattern recognition receptors	Innate	Several types with ability to bind components of different microorganisms: (i) toll: 11 classes: Affinity for different agents (ii) NOD: Bacterial infections (iii) CLEC: Mannose receptors (candida) and mannose-binding lectin (bacteria and fungi) (iv) RIG1: Virus

ADA, adenosine deaminase; BTK, Bruton's tyrosine kinase; CLEC, C-type lectin; Ig, immunoglobulins; JAK3, Janus Kinase 3; NK, natural killer; NOD, nucleotide oligomerization domain; PIDs, primary immunodeficiency; RAG, recombination activating gene; RIG1, retinoic acid-inducible gene I; SLE, Systemic lupus erythematosus.

## Abbreviations

AL: Light chain (AL) amyloidosis
CF: Cystic fibrosis
CT: Computed tomography
CVID: Common variable immunodeficiency
GLILD: Granulomatous-lymphocytic interstitial lung disease
Ig: Immunoglobulins
IgG4-RD: IgG4-related disease
ILD: Interstitial lung disease
PHDs: Primary humoral deficiencies
PIDs: Primary immunodeficiencies
SAA: Serum amyloidosis
SIgAD: Selective IgA deficiency.
